# Mercury and antimony in wastewater: fate and treatment

**DOI:** 10.1007/s11270-016-2756-8

**Published:** 2016-02-23

**Authors:** Andrew J. Hargreaves, Peter Vale, Jonathan Whelan, Carlos Constantino, Gabriela Dotro, Elise Cartmell

**Affiliations:** Cranfield Water Science Institute, Cranfield University, College Road, Cranfield, Bedford, MK43 0AL UK; Severn Trent Water, 2 St John’s Street, Coventry, CV1 2LZ UK; Strategic Advisory Services, Atkins, Chilbrook Oasis Business Park, Eynsham, Oxford, OX29 4AH UK

**Keywords:** Mercury, Antimony, Precipitation, Adsorption, Wastewater, Kepner Tregoe

## Abstract

It is important to understand the fate of Hg and Sb within the wastewater treatment process so as to examine potential treatment options and to ensure compliance with regulatory standards. The fate of Hg and Sb was investigated for an activated sludge process treatment works in the UK. Relatively high crude values (Hg 0.092 μg/L, Sb 1.73 μg/L) were observed at the works, whilst low removal rates within the primary (Hg 52.2 %, Sb 16.3 %) and secondary treatment stages (Hg 29.5 %, Sb −28.9 %) resulted in final effluent concentrations of 0.031 μg/L for Hg and 2.04 μg/L for Sb. Removal of Hg was positively correlated with suspended solids (SS) and chemical oxygen demand (COD) removal, whilst Sb was negatively correlated. Elevated final effluent Sb concentrations compared with crude values were postulated and were suggested to result from Sb present in returned sludge liquors. Kepner Tregoe (KT) analysis was applied to identify suitable treatment technologies. For Hg, chemical techniques (specifically precipitation) were found to be the most suitable whilst for Sb, adsorption (using granulated ferric hydroxide) was deemed most appropriate. Operational solutions, such as lengthening hydraulic retention time, and treatment technologies deployed on sludge liquors were also reviewed but were not feasible for implementation at the works.

## Introduction

Wastewater treatment works (WWTWs) receive metal inputs from both domestic and industrial sources; therefore, discharges from WWTWs have the capacity to elevate metal concentrations in rivers such that harm may occur (Stumm and Morgan 2012). Whereas metals such as copper and zinc have been the subject of numerous studies (Chipasa 2003; Beck and Birch 2012; El Khatib et al. 2012), trace metals such as mercury (Hg) and antimony (Sb) are not monitored on a regular basis (Choubert et al. 2011). Nevertheless, they have been observed throughout the various stages of the wastewater treatment process (Yoshida et al. 2013).

There are strong regulatory drivers that require Hg and Sb removal as part of the wastewater treatment process. Mercury is classified as a priority hazardous substance (PHS) under the Water Framework Directive (WFD) (2000/60/EC) requiring emission cessation. The WFD currently requires that Hg concentrations do not exceed 0.05 μg/L as an annual average (AA) and 0.07 μg/L as a maximum allowable concentration (MAC) in inland surface waters. In the USA, in accordance with the Clean Water Act, national recommended water quality criteria outline standards for the protection of aquatic life and human health in surface water. For Hg, the criterion continuous concentration (CCC) is 0.77 μg/L and the criteria maximum concentration (CMC) is 1.4 μg/L, whilst Sb concentrations may not exceed 5.6 μg/L.

In the UK, the concentration of Sb in drinking water may not exceed 5 μg/L (DEFRA 2015). A combination of factors such as low effluent dilution capacity and that drinking water abstraction locations are often located downstream of WWTW discharges, mean WWTW operators seek to reduce the concentration of Sb in effluent.

Mercury enters wastewater from a variety of sources including dental practice wastes, which can contribute up to 50 % crude Hg concentrations (Bender 2008), fertilisers, landfill leachate, paints, domestic waste inputs, groundwater infiltration, stormwater drainage contributions and historical sources of Hg (Gbondo-Tugbawa et al. 2010; Wang et al. 2004). External and tankered sludge inputs have also been found to influence metal concentrations within the wastewater treatment process potentially increasing metal content, including Hg, within final effluent discharges (Grady Jr. et al. 2012). Antimony concentrations at WWTWs are predominantly associated with its use as a flame retardant in consumer electronics (van Velzen et al. 1998). Other sources of Sb include paints and landfill leachate, which Cyr et al. (1987) reported may contain concentrations in the region of 10 μg/L.

Although there is a need to enhance the removal of these pollutants, an understanding of their fate within WWTWs is limited (Rogers et al. 1996). Indeed, studies into the fate of Sb within WWTWs are rare and existing data focuses on Sb behaviour within natural aquatic systems (Filella et al. 2002). Although the concentrations of Hg in influent and effluent as well as treatment process removal efficiencies have been assessed (Goldstone and Lester 1991; Rule et al. 2006), Hg fate throughout WWTWs is seldom discussed.

Some information on technologies that may be suitable to treat these metals is available. Physicochemical techniques have been considered as potential treatment options (Guo et al. 2009; Ungureanu et al. 2015), whilst membrane filtration has also been deployed for Hg and Sb removal (Chiarle et al. 2000; Kang et al. 2000). The feasibility of specific technologies has not, however, been assessed in the context of future metal management at WWTWs.

This study assess the fate of Hg and Sb and examines the influence of different treatment stages on the overall removal efficiency at a WWTWs. Operational solutions, such as lengthening sludge retention time (SRT), and technologies available for Hg and Sb treatment are also reviewed.

## Materials and Methods

### Study Site

**Fig. 1:**
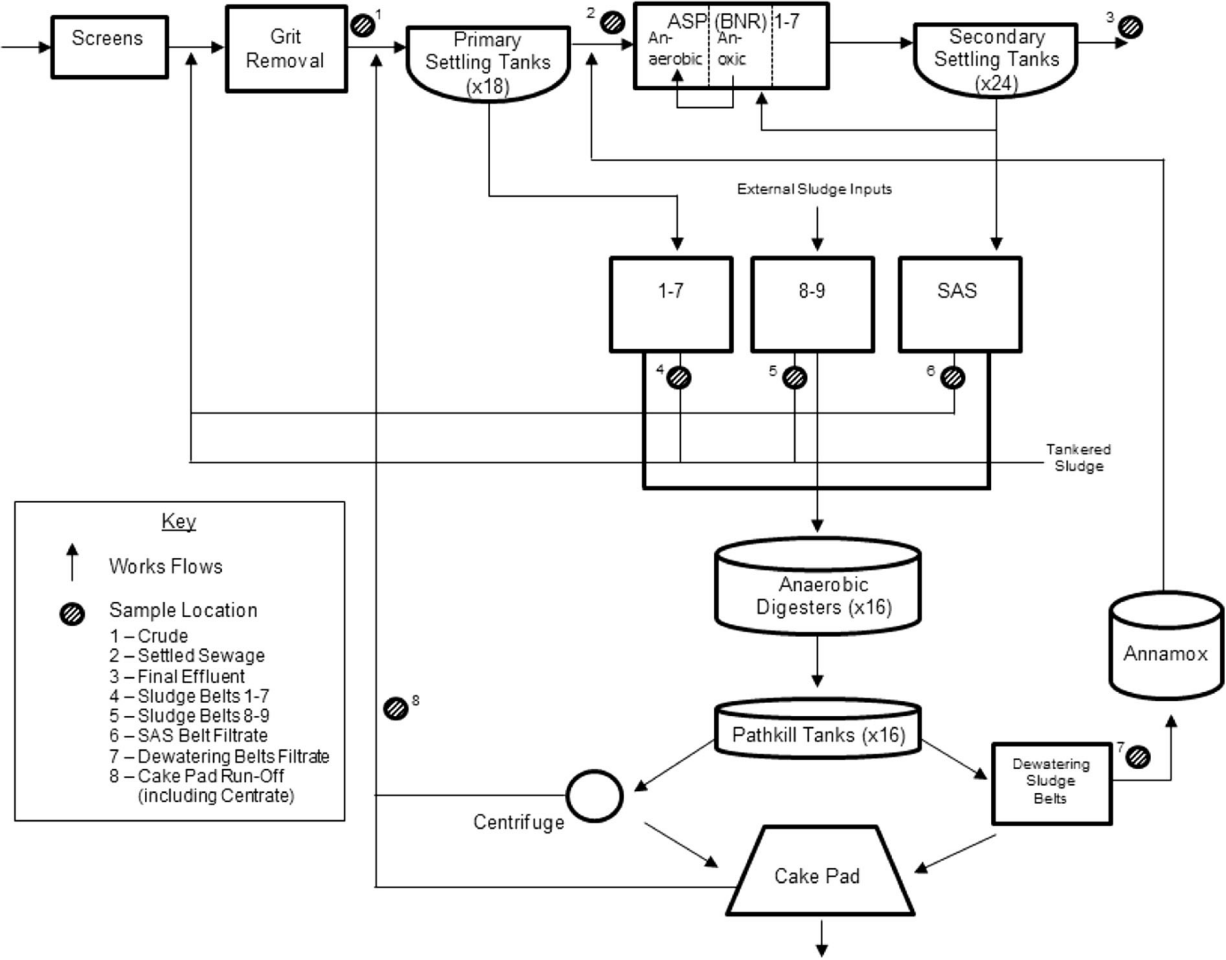
Simplified flow sheet for the site studied including sample locations

The WWTWs examined in this study are located in the UK and utilise the activated sludge process (ASP) treatment technology. The site receives wastewater from a large urban catchment population. The site also accepts external site sludge inputs and domestic waste contributions. A schematic diagram showing the arrangement of treatment processes at the site is provided in Fig. 1. Crude sewage is initially subject to screening and grit removal processes. Wastewater is treated within primary settling tanks followed by activated sludge treatment, consisting of seven lines which operate in a biological nutrient removal (BNR) configuration (containing anaerobic, anoxic and oxic phases). Primary and secondary sludge are thickened separately on sludge belts 1–7 and the surplus activated sludge (SAS) belt, respectively, whilst external sludge inputs enter sludge belts 8–9. Sludge is then treated using anaerobic digestion (AD) and is moved into pathkill (secondary digestion) tanks, after which the sludge is dewatered and stored on a cake pad.

### Sample Collection

Mercury and Sb concentrations were measured across the works from November 2013 to March 2015 at a number of treatment stages identified in Fig. 1, namely crude (1), settled sewage (2), final effluent (3), sludge belts 1–7 (4) and 8–9 (5), SAS belt filtrate (6), dewatering belts filtrate (7) and cake pad run-off (including centrate) (8). A minimum of four grab samples were taken from each location every month. Settled sewage and SAS belt filtrate samples were collected intermittently.

### Analytical Methods

Using standard vacuum filtration equipment and following APHA (2005) procedures, suspended solid (SS) concentrations were determined, whilst total chemical oxygen demand (COD), phosphate (PO_4_) and ammonical nitrogen (NH_4_) concentrations were determined using cell test kits (Fisher Scientific, Leicestershire, UK Ltd). After acidification with nitric acid (HNO_3_), samples were analysed for Hg and Sb content using an ELAN 9000 inductively coupled plasma-mass spectrometer (Perkin Elmer, Beaconsfield, UK).

### Data Analysis

Removal of SS, COD, PO_4_ and NH_4_ was calculated at each treatment stage and throughout the works as a whole. Analysis of these sanitary determinands was undertaken to ensure that the treatment works was operating as expected. Overall and individual treatment stage removal efficiencies were also calculated for Hg and Sb. The dataset was collated into seasonal periods comprising of spring (March, April, May), summer (June, July, August), autumn (September, October, November) and winter (December, January, February) to identify patterns in Hg and Sb inputs and/or works performance and, in particular, to assess the significance of metal mobilisation by rainfall on crude and effluent concentrations. Data outliers and anomalous values were identified and extracted from the dataset using the Grubbs Test (Grubbs 1950). One-way ANOVA tests were used for statistical analysis which considered the significance, with the threshold *p* ≤ 0.05, of year on Hg and Sb concentrations found throughout the works, whilst the influence of seasonality on removal efficiency and the effectiveness of treatment stages were also assessed. Pearson’s correlation coefficient tests were used to identify trends between sanitary determinand and metal removal and to identify if rainfall (using Met Office data) influenced Hg and Sb concentrations found in crude and effluent samples.

### Kepner Tregoe Analysis

The treatment technologies available to enhance the removal of Hg and Sb were identified from a review of literature with a screening process employed to select the candidate technologies. Screening involved the elimination of technologies unable to remove Hg and Sb to concentrations below UK (Hg <0.05 μg/L, Sb <5 μg/L) and USA (Hg 0.77 μg/L, Sb 5.6 μg/L) standards. In order to evaluate the screened technologies and select the most suitable to remove Hg and Sb from wastewater, a Kepner Tregoe (KT) analysis was undertaken (Kepner Tregoe 2013). Kepner Tregoe is a decision-making approach, which utilises selection indicators and assigned weightings, and incorporates a point scoring system enabling technologies to be compared in relation to their suitability. The selection indicators and weightings chosen (Table 1) represent the most important considerations for the wastewater treatment operator (based on feedback from wastewater treatment specialists *n* = 10), with studies at WWTWs with equivalent research conditions (e.g. metal concentrations) utilised to inform treatment technology selection.

**Table 1:** Kepner Tregoe selection indicators, their weightings and associated operational criteria

Selection indicators	Operational criteria	Weighting
Effectiveness for concentration range	The technology can achieve by itself the final Hg and Sb requirements	10
Footprint	It is compact and/or can be retrofitted into the existing works	8
CAPEX	Costs and feasibility of construction	6
Energy consumption	Energy requirements for technology usage	7
Maintenance requirements	Costs of continual operation	6
Chemical usage	Quantity, diversity and hazardous nature of chemical usage	5
Ready to use	Proven to work on wastewater at full scale anywhere in the world	5

## Results

### Works Operating Conditions

**Fig. 2:**
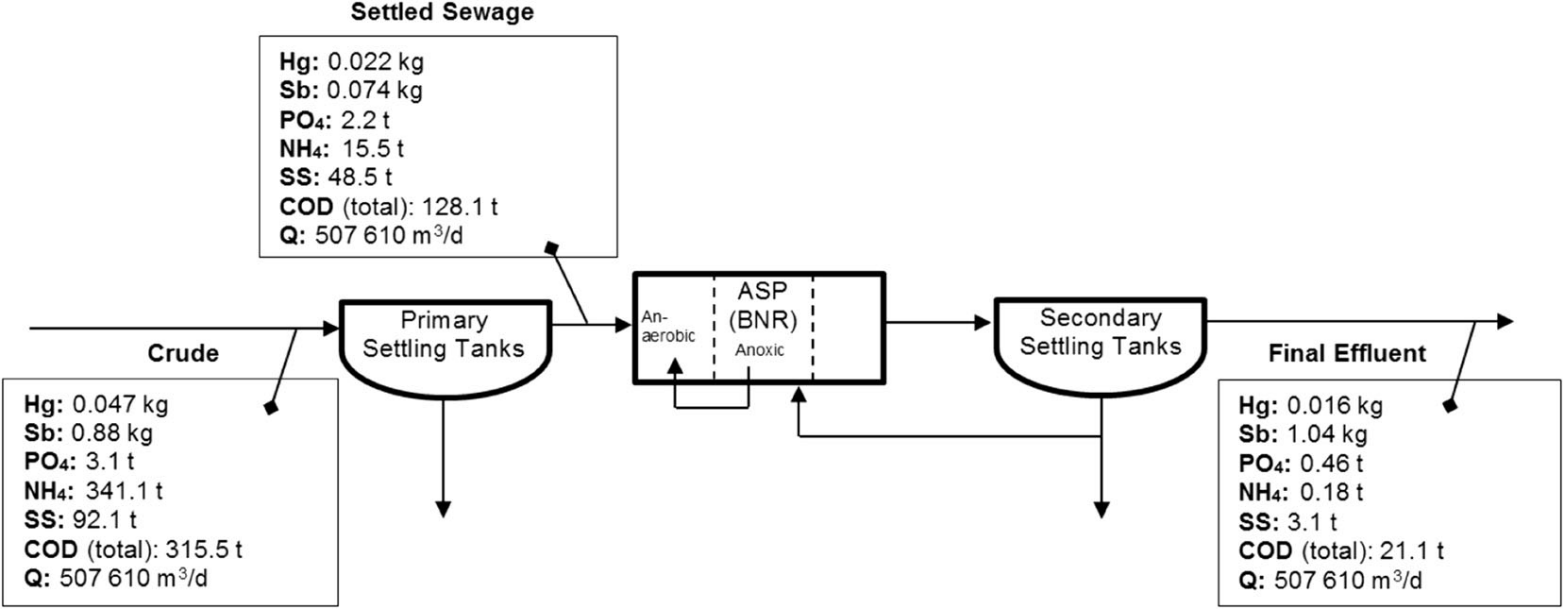
Mass flux calculated for the works

The calculated removal efficiencies for NH_4_ (99.9 %), SS (96.6 %), COD (93.6 %) and PO_4_ (84.7 %) indicated that the treatment works operating conditions were satisfactory (Table 2). Analysis of flow rate data also found that seasonality had no significant influence (*p* = 0.453) on inflows at the works. Therefore, the average flow rate (507,610 m^3^/day) was used within mass flux calculations (Fig. 2).

**Table 2:** Removal efficiencies for the works for mercury and antimony and sanitary determinands

Removal efficiency (%)	Hg	Sb	PO_4_	NH_4_	SS	COD
Primary removal	52.2	16.3	28.2	95.5	47.4	59.4
Secondary removal	29.5	−28.9	78.6	98.8	93.6	84.2
Overall REMOVAL	66.3	−15.2	84.7	99.9	96.6	93.6

### Mercury and Antimony Removal

The overall removal efficiency for Hg (66.3 %) was low compared with values recorded for sanitary determinands (84.7–99.9 %), with substantially lower Hg removal recorded for the secondary treatment stage (29.5 %) in comparison with the primary treatment stage (52.2 %). Concentrations recorded at sample locations 1–3 (Fig. 1) for Hg at the works were not statistically significant (*p* = 0.08), with a small change between mean crude (0.092 μg/L) and final effluent values (0.031 μg/L) and little difference between mean settled sewage (0.044 μg/L) and final effluent concentrations (0.031 μg/L), which ranged from 0.01 to 0.06 μg/L. The final effluent concentrations were therefore below the current MAC-environmental quality standard (EQS) (0.07 μg/L) and AA-EQS (0.05 μg/L).

**Fig. 3:**
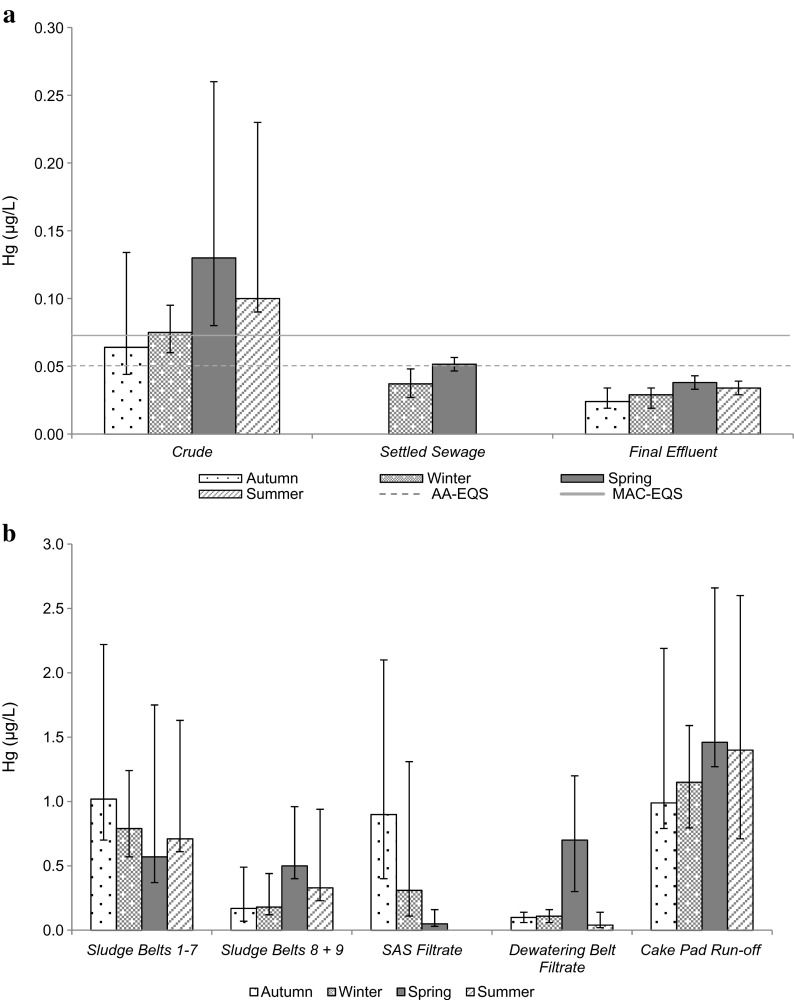
Hg concentrations observed in effluent (**a**) and sludge samples (**b**) at the works (mean ± SD) in relation to the current AA-EQS and MAC-EQS

Mean concentrations of Hg within the cake pad run-off (1.25 μg/L) were high compared with sludge belts 1–7 (0.77 μg/L), sludge belts 8–9 (0.31 μg/L), SAS belt filtrate (0.42 μg/L) and dewatering belts filtrate (0.24 μg/L). However, the large variability of Hg concentrations observed for each sludge sample location at the works (Fig. 3b) meant that the differences between sample points were not statistically significant (*p* = 0.494).

A significant positive correlation between SS and Hg removal was observed for the works (*r* = 0.514; *p* < 0.001), with a statistically significant, albeit small, positive correlation between Hg and COD removal (*r* = 0.189; *p* = 0.02). These correlations confirm that removing SS will improve Hg removal at the works.

For Sb, although some removal occurred at the primary treatment stage (16.3 %), negative removal was recorded across secondary treatment (−28.9 %) with an overall removal efficiency of −15.2 %, indicating that Sb concentrations increased throughout the works. This observation was reflected within recorded concentrations which showed little change between crude (1.73 μg/L) and settled sewage values (1.45 μg/L), whilst Sb concentrations were enhanced in final effluent (2.04 μg/L) (Table 3). Differences between these sample points were not statistically significant (*p* = 0.217). Furthermore, the coefficient of variation (CoV) demonstrates an increase in variability across the works treatment stages; similar values for crude (0.40) and settled sewage (0.41) were calculated, whilst a substantially higher value was observed for final effluent (0.68).

**Table 3:** Sanitary determinand and metal concentrations observed in crude, settled sewage and final effluent samples at the works (mean ± SD)

Determinand	Crude	Settled sewage	Final effluent
SS (mg/L)	177.11 (±79)	92.52 (±32)	6.08 (±1.5)
PO_4_ (mg/L)	5.93 (±1.6)	4.26 (±0.7)	0.91 (±0.2)
NH_4_ (mg/L)	671.41 (±264)	30.51 (±8.22)	0.36 (±0.4)
COD (mg/L)	621.61 (±160)	252.41 (±51)	40.02 (±7.8)
Hg (μg/L)	0.092 (±0.06)	0.044 (±0.03)	0.031 (±0.03)
Sb (μg/L)	1.73 (±0.7)	1.45 (±0.6)	2.04 (±1.4)

**Fig. 4:**
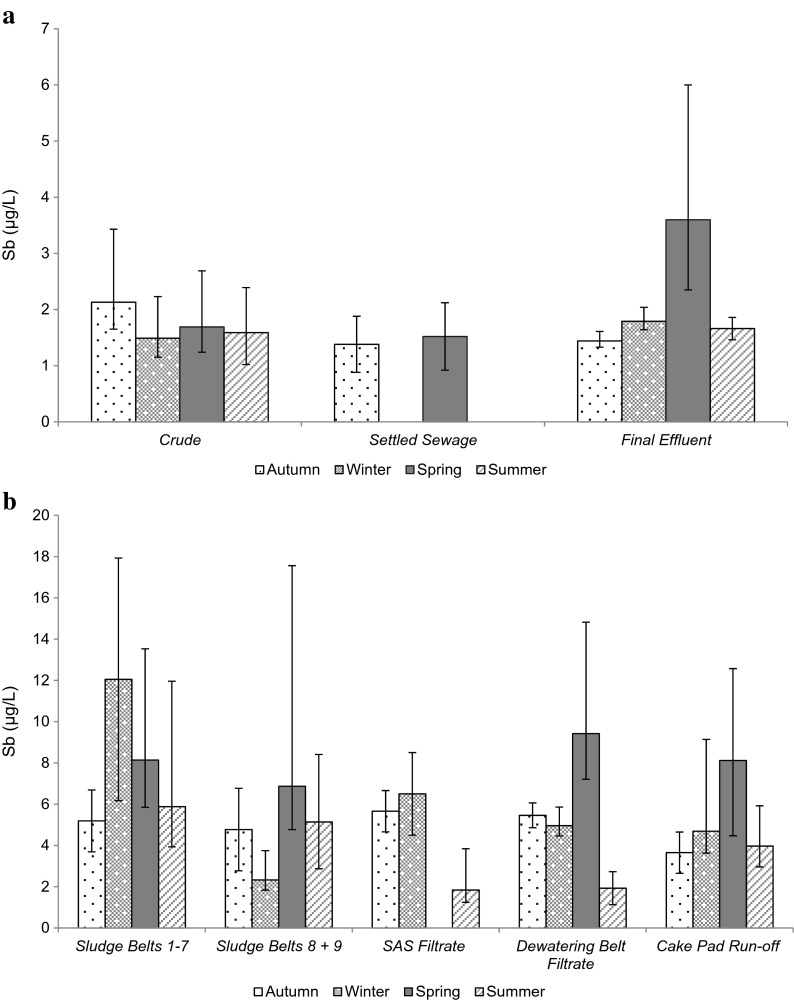
Sb concentrations observed in effluent (**a**) and sludge samples (**b**) at the works (mean ± SD)

Mean concentrations of Sb within sludge belts 1–7 (7.81 μg/L) and sludge belts 8–9 (6.03 μg/L) were high compared with SAS belt filtrate (2.97 μg/L), dewatering belts filtrate (5.08 μg/L) and cake pad run-off (5.11 μg/L). However, the large variability of Sb concentrations observed for each sludge sample location at the works (Fig. 4b) meant that the differences between sample points were not statistically significant (*p* = 0.214).

A statistically significant negative correlation was observed between Sb and SS removal (*r* = 0.653; *p* < 0.001) as well as Sb and COD removal (*r* = 0.545; *p* = 0.004) at the works. These correlations demonstrated that removing SS and COD from the works had little effect on Sb removal.

### Influence of Seasonality on Mercury and Antimony Concentrations

It was initially determined that year had no significant influence on Hg or Sb concentrations found within crude (Hg: *p* = 0.225; Sb: *p* = 0.164), effluent (Hg: *p* = 0.358; Sb: *p* = 0.121) and sludge samples (Hg: *p* = 0.271; Sb: *p* = 0.103), justifying the collation of data into seasons for further analysis.

Differences in the mean, seasonal concentrations recorded at all sludge sample locations were not statistically significant for Hg (*p* = 0.242; Fig. 3b) or Sb (*p* = 0.191; Fig. 4b) at the works. Similarly, differences in the mean, seasonal Hg concentrations recorded for crude, settled sewage and final effluent samples were not statistically significant (*p* = 0.271; Fig. 3a). However, as demonstrated in Fig. 4a, significant seasonal variation was present for Sb concentrations observed in final effluent (*p* = 0.037) with further analysis determining that spring Sb concentrations were higher than those recorded for other seasons.

No statistically significant correlation was observed between rainfall and Hg (*r* = 0.09; *p* = 0.168), and rainfall and Sb concentrations (*r* = 0.06; *p* = 0.205) observed within crude and final effluent samples, indicating that rainfall did not mobilise Hg or Sb into the works or influence the effectiveness of wastewater treatment.

### Technology Selection to Enhance Mercury and Antimony Removal

**Fig. 5:**
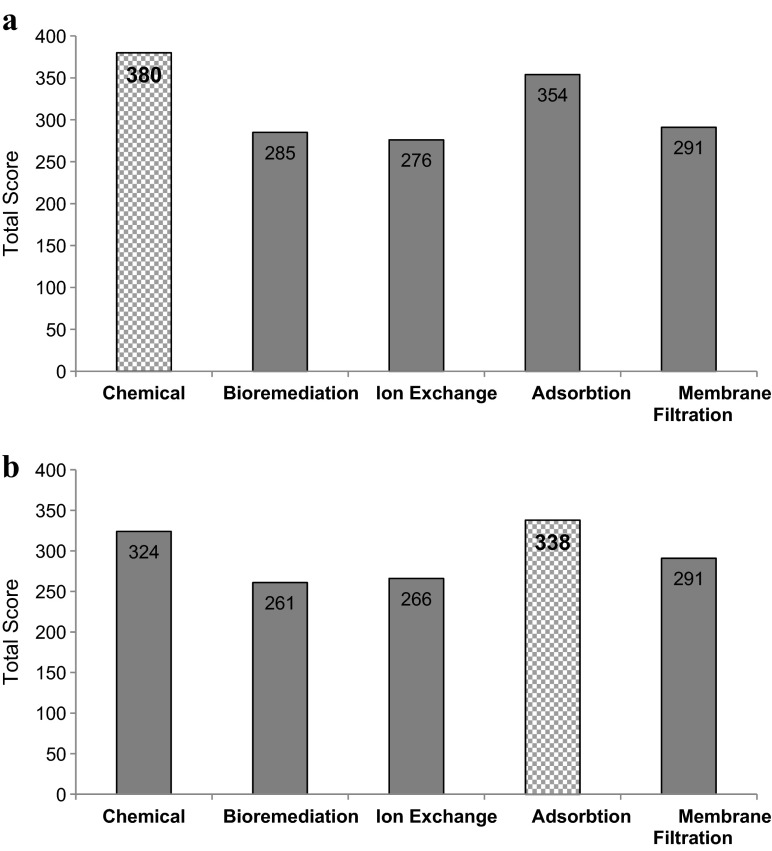
Results of Kepner Tregoe analysis on technologies for the removal of Hg (**a**) or Sb (**b**) from effluent streams at the works

The initial screening process highlighted that membrane filtration, bioremediation, adsorption, chemical and ion exchange technologies (Table 4) could be used to remove Hg and Sb to below UK (Hg 0.05 μg/L, Sb 5 μg/L) and USA (Hg 0.77 μg/L, Sb 5.6 μg/L) standards. The results of the KT analyses, which assessed the suitability of current effluent treatment technologies for implementation at the works, are presented in Fig. 5. Chemical techniques were determined as most appropriate to enhance Hg removal at the works (Fig. 5a) as this low-cost option can treat large volumes of wastewater, is effective over a wide pH range and exhibits operation simplicity (Karman et al. 2015; Ungureanu et al. 2015). Adsorption was deemed most suitable to increase Sb removal (Fig. 5b) as a variety of adsorbents have been used to remove Sb with high removal efficiencies reported (85–99 %) (Guo et al. 2009).

**Table 4:** Summary of technologies capable of removing Hg and Sb to concentrations below UK (Hg 0.05 μg/L, Sb 5 μg/L) and USA (Hg 0.77 μg/L, Sb 5.6 μg/L) standards

Technology classification	Treatment outline	Advantages	Limitations	Factors influencing removal efficiency
Adsorption Synthetic adsorbent biosorbents	Adsorption involves adhesion of ions, atoms or molecules from dissolved solid, liquid or gaseous phases to a surface (Stumm and Morgan 2012). Adsorption can be a reversible mechanism, allowing adsorbent regeneration through desorption processes (Fu and Wang 2011)	Cost-effective, design flexibility, produces high quality effluent	Contaminant sensitivity-fouling and plugging, spent media disposal	Contaminant concentration, pH, flow rate, fouling, spent media
Bioremediation Bioreactors	Bioreactors are vessels in which chemical processes occur, involving organisms directly or organism-derived biochemically active substances. A supply of chemically inert free-flowing medium, which acts as a receptacle, allows bacterial breakdown of sewage. This mechanism can be aerobic or anaerobic (Grady Jr et al. 2012)	Continuous operation, temperature control, simple construction	Pre-treatment requirements, solids disposal	pH, temperature, available nutrients, contaminant concentration
Chemical Precipitation Coagulation/flocculation	Precipitation involves chemical reactions with heavy metal ions forming insoluble precipitates, which are extracted from water using sedimentation and filtration techniques (Fu and Wang 2011). Coagulation involves wastewater charge neutralisation and the formation of a mass which is a sufficient size to be trapped through filtration (Mazille 2015). Flocculation defines the stirring/agitation procedure applied to encourage particle agglomeration (Mazille 2015)	Simple operation, low capital cost	Sludge generation, sludge disposal cost	Other compound presence, pH, chemical dosage, sludge disposal
Membrane filtration Ultrafiltration (UF) Reverse osmosis (RO) Nanofiltration (NF)	UF works at low transmembrane pressures for the removal of colloidal and dissolved material (Ersahin et al. 2012). RO uses a semi-permeable membrane, which allows fluid being purified to go through, whilst rejecting contaminants. RO has high pumping pressures and requires the restoration of membranes. NF is an intermediate process between RO and UF, in which benefits occur from ease of operation and reliability, whilst comparatively low energy consumption and greater efficiency are also achieved (Ersahin et al. 2012)	High separation selectivity, small space requirement	Membrane fouling, high energy/ operational cost	Contaminant concentration, molecular weight of contaminants/solids, characteristics of untreated water
Ion exchange	Ion exchange resins, whether they are naturally solid or synthetic, instigate cation exchange with metals in wastewater. Synthetic resins are predominantly used due to their reported higher efficiency, whilst commonly used cation exchangers are strongly acidic resins with sulphonic acid groups or weak acid resins with carboxylic acid groups (Gode and Pehlivan 2006)	Low time consumption, no sludge generation	High capital cost, only some resins suitable for metal removal	pH, temperature, contact time, initial metal concentration, ionic charge

## Discussion

### Mercury and Antimony Occurrence Throughout Wastewater Treatment

Mean crude Hg concentrations at the works (0.092 μg/L) are higher than the average value (0.066 μg/L) recorded for 16 WWTWs throughout the UK (Gardner et al. 2013). The value for the works is however less than mean crude concentrations (0.5 μg/L) observed by Rule et al. (2006) for UK WWTWs, whilst large industrial inputs have led to values as high as 1.6 μg/L observed in crude sewage (Lester 1987). From a global perspective, Ribeirao Preto WWTWs (Brazil) serves over 1000 diverse industrial activities and although the majority of these are defined as small businesses (chemical, medical and food industries), many contribute to the 0.1 μg/L Hg concentration observed in crude samples (Oliveria et al. 2007). A mean crude Hg concentration of 0.1 μg/L was also recorded at Henriksdal WWTWs (Sweden) with dental inputs recognised as having a prominent contribution (Sorme and Lagerkvist 2002). Dental wastes, more specifically amalgam, were previously found to be responsible for 53 % of global Hg emissions (WHO 2013), with up to a 50 % contribution to crude concentrations observed at WWTWs (Bender 2008). However, Hg has been subject to source control with the Minamata Convention treaty, agreed by the United Nations Environment Programme (UNEP) in 2013, stating that progress must be made to reduce the use of mercury in dentistry, encouraging use of non-mercury containing alternatives (UNEP 2013). Consequently, high influent concentrations of Hg at WWTWs in the present day often refer to wastewater infiltrated with landfill leachate and/or agricultural chemicals such as fertilisers (Wang et al. 2004). From a locational perspective, the site studied is not in the vicinity of landfill or agricultural operations, predominantly receiving industrial influent. It is therefore suggested that relatively low crude concentrations of Hg at the works, compared with findings by Rule et al. (2006), has resulted from dentistry practice changes, reflecting expected findings for global WWTWs (Mackey et al. 2014).

The relatively high mean crude Hg concentration (0.092 μg/L) observed at the works in comparison to post Minamata Convention values at WWTWs in the UK (0.066 μg/L; Gardner et al. 2013) was not suggested to be unusual, as the value recorded was still within the range reported in the CIP programme (Gardner et al. 2013). However, high crude concentrations of Sb compared with typical concentrations (0.2–0.4 μg/L) outlined by Choubert et al. (2011) were associated with the high industrial inputs at the works. These inputs contain high Sb concentrations amid contributions from consumer electronics and paints which have ever-increasing obsolete characteristics (van Velzen et al. 1998).

### Mercury Fate and Removal Throughout Wastewater Treatment

Mercury fate and removal throughout the wastewater treatment process is influenced by interactions of this metal with SS and organic compounds (Vernon and Bonzongo 2014). Mercury has a strong association with both these components, and this relates to high sorption characteristics of this metal (McKay et al. 1989). This attribute is demonstrated using the metal partition coefficient (K_d_), which is the ratio of sorbed metal concentration to dissolved metal concentration at equilibrium (Allison and Allison 2005). For Hg, surface water studies have found relatively high K_d_ values for partitioning with suspended solids (4.7) and dissolved organic carbon (DOC) (5.4), demonstrating that mercury portrays high affinity for both organic and inorganic particulate matter (Allison and Allison 2005; Bravo et al. 2011). Although studies which have calculated K_d_ values for Hg in wastewater are limited, Hg has been found to demonstrate high affinity for SS and organic compounds within WWTWs (Goldstone et al. 1990; Wagner-Döbler et al. 2000). This is reflected at the site studied with significant positive correlations found between Hg and SS removal as well as Hg and COD removal. Removal of Hg within primary treatment at the site is relatively low (52.2 %) compared with the global average (62 %) recorded by Ziolko et al. (2011), however, shows a similar efficiency to findings in Canada (54 %; Oliver and Cosgrove 1974) and the USA (57 %; Barth et al. 1965). This suggests that primary treatment has satisfactory Hg removal at the works, but removal increases are achievable through operational solutions such as lengthening HRT (Petrie et al. 2014).

Reduced Hg removal within secondary treatment (29.5 %) compared with primary treatment (52.2 %) at the works (Fig. 2) is expected and is suggested to result from metal accumulation onto biosolids, due to the recirculation of returned activated sludge (RAS) (Fig. 1). This process enhances metal content per mass of solids and subsequently concentrations found in effluent (Huang and Wang 2001). Chen et al. (1974) determined that within biological treatment 45–55 % of Hg present was associated with settleable particulates, subsequently stating that Hg is removed by adsorption onto bacterial solids. Physicochemical adsorption of soluble Hg to activated sludge has also been documented (Wu and Hilger 1985) with Hg complexes (with organic compounds) adsorbing onto the surface of SS or microbiological communities (Guo et al. 2009). This is reflected within the significant positive correlation found between Hg and SS removal at the works.

Secondary treatment Hg removal at the works (29.5 %) was lower than the global average for biological treatment at ASP works (68 %) (Ziolko et al. 2011), whilst UK ASP works, such as Whitlingham WWTWs (Norwich, England), demonstrated mean removal of 47 % (Goldstone et al. 1990). Within secondary treatment at the works, a lower removal efficiency for Hg compared with primary treatment coincided with SS and COD removal increases (Table 2). These findings are reflected within a works studied by Goldstone et al. (1990), who found that this resulted from high Hg concentrations entering the treatment process from returned sludge liquors. Sludge returns prevent dissolved Hg from attaching to the surface of particles, therefore enhancing final effluent metal concentrations (Goldstone et al. 1990). This is suggested to reason why low removal efficiency was seen for secondary treatment at the works studied. More specifically, cake pad run-off is suggested to be the prominent influence as this return has consistently higher mean Hg concentrations compared with other sludge sampling locations throughout all seasons studied (Fig. 3b). Furthermore, as the flow sheet suggests (Fig. 1), this return is not accounted for within the crude samples collected and analysed at the site.

Despite specific sludge return attribution, it must be considered, however, that all sludge samples contained Hg (Fig. 3b) with wide ranging concentrations observed for all locations at the works (Fig. 3b). These inputs can inhibit site operations by reducing sludge settleability; however, this has not occurred at the site with a satisfactory primary tank performance observed (Table 2). Nevertheless, increasing Hg concentrations within the works, which all sludge returns contribute to, reduces metal removal elevating final effluent concentrations, with a correlation existing between increasing metal concentration and decreasing metal removal (Chipasa 2003).

### Antimony Fate and Removal Throughout Wastewater Treatment

Due to the typical low abundance of Sb in wastewater (0.2–0.4 μg/L) (Choubert et al. 2011), Sb wastewater studies have been largely neglected to date, and therefore, there is a lack of existing data on Sb partitioning in WWTWs. However, published data on Sb partition between solid and dissolved phases in surface waters clearly indicates that Sb is almost exclusively present in the dissolved phase (Jarvie et al. 2000; Filella et al. 2002) with K_d_ values as low as 2.7 reported for Sb in surface waters (Allison and Allison 2005). Tanizaki et al. (1992) also reported that in river water, 90 % Sb was in the soluble phase (<0.45 μm) with further fractionation analysis of this phase identifying that 70 % of soluble Sb was associated with colloidal matter of <500 Da in diameter. Although it is recognised that surface waters contain different particulate species to those found in wastewater, the pH range (pH 7–9) is equivalent; therefore, Sb is anticipated to have a high solubility in wastewater regardless of its oxidation state (Filella et al. 2002). Weak interactions between Sb and suspended particles are anticipated to exist in wastewater (Filela et al. 2002), and this is supported by the significant negative correlation observed between Sb and SS removal at the studied works and the low efficiency (16.3 %) observed for Sb throughout primary treatment at the works.

Thermodynamic analysis attests that Sb exists as Sb(V) in oxic and Sb(III) in anoxic environments (Fillela et al. 2002), and despite limited systematic studies existing on this aspect, it has been determined that Sb within wastewater treatment is unstable (Sun et al. 1982). Instability is prominent within the ASP amid fluctuating anaerobic and anoxic conditions (Fig. 1). The high solubility and instability characteristics of metals, such as Sb, are correlated with low, if any, removal throughout activated sludge treatment (Brown et al. 1973). This relates to the low complexation capacity of these metals and subsequently a low affinity for activated sludge biomass (Lester 1983). Whilst the availability of metals, such as lead (Pb), readily form stable, insoluble complexes with sewage sludges (Brown et al. 1973) are also a contributing factor to low removal. Accounting for these characteristics, it can be suggested that any complexes formed between extracellular polymers and Sb will be weak, as such during activated sludge treatment oxidation of polymers in the ASP is likely to result in Sb release back into effluent. This phenomenon has been observed for cadmium (Cd) and arsenic (Ar) (Lawson et al. 1984) and is subsequently suggested to contribute to the negative Sb removal (−28.9 %) existing within secondary treatment at the works. However, further examination of Sb speciation within wastewater is required to confirm this suggestion.

Increases within Sb concentrations across the works (Table 2), reflected within the overall calculated removal (-15.2 %), were suggested to result from Sb being imported with sludge to the works. Antimony within imports was suggested to predominantly exist within the free ionic form, demonstrating a low sorption potential and little affinity for SS (Fillela et al. 2002). This finding was reflected within the significant negative correlation between Sb and SS removal at the works. Returned sludge liquors, which demonstrated wide ranging Sb concentrations for all sludge sample locations (Fig. 4b), were therefore suggested to contribute to the variable and elevated final effluent concentrations at the site (Table 2).

### Operational Solutions to Enhance Mercury and Antimony Removal

Most effective operational solutions to enhance metal removal during primary settlement relate to lengthening HRT, with metal concentrations found in settled sewage reduced with increasing HRT (Petrie et al. 2014). This results from high proportions of metals, including Hg, in crude sewage being associated with particulates (Wang et al. 2014). Enhancements to HRT in most instances, including at the site studied, would require infrastructural developments as sufficient space is not present with onsite storm tanks and remote holding tanks are unavailable to act as counterbalances for fluctuating sludge flows (Petrie et al. 2014). In addition, enhanced HRT requires improved primary treatment through enhanced particulate bound metal removal to prevent solubilisation within secondary treatment (Kumar et al. 2014). An alternative to increasing primary settlement tank sizes involves the use of micro-screens which are determined to improve SS removal with a comparatively reduced footprint (Unger and Brinker 2013).

Metal removal within biological (secondary) treatment that incorporates activated sludge matrices relies entirely on partitioning (Ziolko et al. 2011). This is dictated by three processes; physical entrapment of insoluble metals, binding of soluble metals to extracellular polymers/bacterial walls and active cellular uptake by bacterial cells (Ziolko et al. 2011). Partitioning is the main removal pathway for metals amid increased concentrations observed in the particulate phase of returned activated sludge at each sludge retention time (SRT) increase (Petrie et al. 2014). Total metal removal is therefore correlated with enhanced SRT amid activated sludge comprising of smaller flocs, increasing floc surface area and subsequently binding site availability (Stoveland and Lester 1980; Petrie et al. 2014). Enhancing SRT instigates decreases in effluent SS and COD whilst increasing sludge volume (Stoveland and Lester 1980). Consistent SRT is therefore desired at the works to develop better process control and continuity. This can be achieved operationally by using in situ SS probes and real-time flow measurements. It must be considered that increased SRT is correlated with greater aeration demands and subsequently costs (Amanatidou et al. 2015). Although it has been shown by Leu et al. (2012) that higher SRT achieves improved oxygen transfer due to smaller and more uniform activated sludge flocs, it has been found that Hg concentrations are unaffected by SRT (Conklin et al. 2007).

Significant variations within sewage flows at the works (16,488–96,274 m^3^/day) results in a dynamic system that experiences significant variation within SRT and HRT (Petrie et al. 2014). The inability to feasibly enhance HRT and increasing costs associated with lengthened SRT means that operational solutions are not sustainable options for Hg and Sb management at the site studied. Consequently, metal removal technologies need to be considered for additional treatment.

### Technologies for Sludge Liquor Treatment

Heavy metals, such as Hg, in sludge liquors are suggested to adhere to microorganism cell surfaces (Yoshizaki and Tomida 2000), therefore enabling potential technology implementations to remove metals existing on these surfaces (Volesky 1990).

Biopolymers existing within sludge have low affinity characteristics, emitting buffer action toward inorganic acids (Yoshizaki and Tomida 2000), rendering them ineffective for Hg removal. However, research conducted on diverse acid types determined that phosphoric acid (42.5 %) instigates efficient Hg removal (100 %) (Yoshizaki and Tomida 2000) on a laboratory scale. Phosphoric acid demonstrates high affinity for biopolymers, therefore can easily make contact with metals that exist in the vicinity, effectively dissolving them (Yoshizaki and Tomida 2000). In addition, heavy metals in phosphoric acid solutions (after extraction) are removed with ease by cation exchange resin processing (Yoshizaki and Tomida 2000). It must be considered that the use of phosphoric acid of this concentration (42.5 %) would exhibit unsustainable chemical usage and financial costs. Reduced concentrations lead to reduced removal efficiencies with phosphoric acid (8.5 %) demonstrating 46 % removal (Yoshizaki and Tomida 2000). This trend supports the findings of Stylianou et al. (2007) who state precipitation removal efficiency is enhanced following increases in acid concentration, temperature and contact time. Inflation of these parameters results in an enhancement of the offered energy for the breakdown of chemical bonds of metals found in sludge (Stylianou et al. 2007).

It is acknowledged that biosorbents can remove metals from sludge by ion exchange and adsorption (Hu et al. 2015). Both these mechanisms are influenced by biosorbent components and operational conditions with lengthened HRT and SRT enhancing technique removal efficiency (Stoveland and Lester 1980; Petrie et al. 2014). Ion exchange removal of heavy metals from sewage sludge is however suggested to yield relatively low efficacy (50–60 %), with both gel and macroporous cation exchange resins demonstrating similar effects (De Villiers et al. 1995). Adsorption mechanisms, applied to sewage sludge, are influenced by SS and organic compounds which in most instances cause media fouling and plugging, therefore requiring pre-treatment with flocculation, settling and filtration or oil-water separation (Hu et al. 2015). These limitations alongside the influence of colloids are also responsible for membrane fouling (Fu and Wang 2011). Membrane filtration is therefore suggested to be unfeasible for sludge treatment amid excessive costs and maintenance requirements. Despite adsorption limitations alkali adsorption treatment has been deployed. Alkali treatment is a biosorbent method to enhance sewage sludge adsorption capacity, increasing complexation through the formation of more ionised functional groups (Hu et al. 2015). This technique indicates the following bonding affinity trend for alkali-treated sewage sludge to heavy metals Pb^2+^ > Cd^2+^ > Ni^2+^ (Hu et al. 2015), demonstrating that alkali treatment may effectively remove Hg as this element shows similar binding affinity to Pb (Bailey et al. 1999).

There are currently no studies available which demonstrate the application of technologies to remove Sb from sludge streams at WWTWs. This is suggested to relate to Sb characteristics, amid the low binding affinity of this metal with SS, organic compounds or microorganisms (Fillela et al. 2002). Therefore, despite technologies being available for the removal of metals from sludge liquors, they are not currently commercially available at full scale; therefore, current effluent treatment technologies should be considered.

### Technology Selection: Additional Treatment of Effluent Stream

Bioremediation has a relatively high footprint and to achieve desired concentrations for both Hg and Sb the implementation of buffering tanks are required for wastewater dilution to enable continuous works operation (Wagner-Döbler et al. 2000). To achieve effective removal efficiency, an activated carbon filter should also be used in combination (Wagner-Döbler et al. 2000). Despite relatively low chemical usage and maintenance requirements, bioremediation is foreseen to be an unsustainable option, particularly considering this technology is seldom implemented for Hg and Sb removal (Mani and Kumar 2014). This characteristic is also apparent for ion exchange. Ritter and Bibler (1992) state that Duolite^TM^ GT-73 resin demonstrates reliable removal of Hg and suggest that this resin would effectively remove Sb. Relative uncertainty within the removal ability of synthetic ion exchange resins from effluent, which also include sulphonated polystyrene-divinylbenzene and Amberlite 252 (Monteagudo and Ortiz 2000), has however led to the development of alternative solutions (Wang and Peng 2010). Natural zeolites (naturally occurring silicate minerals) are used for metal removal due to their high abundance and relatively low costs (Karman et al. 2015). Though reported usage of zeolites is becoming more frequented, they are limited at present compared to synthetic resins and are predominantly implemented at laboratory scale (Gode and Pehlivan 2006; Karman et al. 2015). This alongside the observation that ion exchange resins are implicated by initial metal concentrations (Gode and Pehlivan 2006), which for Hg and Sb are low (μg/L ranges), sufficiently decreases potential removal efficiencies achieved (Barakat 2011), therefore reasoning why low total KT scores were attributed to this technology for both Hg (276) and Sb (266) removal (Fig. 5a, b).

Membrane filtration has demonstrated effective removal of Hg from wastewater effluent (Uludag et al. 1997; Chiarle et al. 2000) and effective Sb removal to provide drinking water/high purity water with Sb concentrations in micrograms per litre ranges (Kang et al. 2000). This technology also demonstrates space-saving and easy-operation characteristics (Fu and Wang 2011), however displays low total values within KT analyses (Fig. 5a, b) due to its unsustainable nature. Membrane filtration demonstrates high CAPEX and maintenance requirements (from membrane fouling) (Kang et al. 2000; Fu and Wang 2011) as well as high energy needs, such as adsorption pre-treatment to instigate Hg formation as precipitates. These requirements are necessary to maintain stable operations and obtain higher flux rates which equate to high carbon and financial costs (Wiesner et al. 1994; Fu and Wang 2011).

Adsorption has been deployed for Hg removal with granular activated carbon (GAC) acknowledged to be effective, amid Hg complexes with organic compounds readily adsorbing onto the surface of SS or microbiological communities (Guo et al. 2009). However, regeneration of GAC involves heating to desorb contaminants which release volatile Hg compounds. Further treatment is also necessary before spent media can be disposed, making large-scale application expensive (Sharma et al. 2015). Adsorption techniques therefore demonstrate high-maintenance requirements (spent media disposal), high chemical usage and high CAPEX costs (to implement further treatment). These factors reason why adsorption has a lower total KT score (354) than chemical techniques (380) for Hg removal (Fig. 5a). Chemical techniques, however, have a lower total score for Sb compared with Hg removal, and this is because despite chemical mechanisms being commonly used, they are not specifically designed for Sb removal and therefore exhibit low removal efficiencies (Kang et al. 2003). Guo et al. (2009) did however report 98 % removal of Sb after conducting pH adjustments and dosing with ferric coagulants, which are found to be more effective than alum and lime coagulation (Ungureanu et al. 2015). Precipitation may occur but Sb would have to compete with other compounds found within effluent for sulphates and indeed metals (Guo et al. 2009).

#### Final Selection: Additional Treatment to Enhance Mercury and Antimony Removal

Chemical techniques are most commonly used in metal-removal technologies within effluent streams (Fu and Wang 2011). They are a low-cost option that can treat large volumes of wastewater (Karman et al. 2015). More specifically, sulphide precipitation is found to accomplish outflow Hg concentrations <1 μg L^−1^ without issue, amid the ability of Hg to readily react with sulphide forming mercury sulphide (HgS) (Guo et al. 2009). However, handling of the toxic H_2_S produced requires extensive safety measures and chemical usage (Wagner-Döbler et al. 2000). This limitation is however offset by the ability of chemical techniques to be effective over a wide pH range and exhibit operation simplicity (Ungureanu et al. 2015). These factors demonstrate why the highest total KT score for Hg was attributed to chemical techniques (Fig. 5a). Large metal inputs at the head of the works, particularly from cake pad run-off and sludge belt returns (Fig. 3b), mean that for Hg removal, chemical treatments to crude sewage (including site returns) before it enters primary treatment are the most suitable option. This implementation would raise system pH and precipitate dissolved metals as hydroxides, which would settle out in primary settling tanks before secondary treatment commences (Oliver and Cosgrove 1974). This is suggested to be a distinct advantage of precipitation as deployment of adsorption, and ion exchange would be most appropriate after primary treatment to prevent equipment clogging and prohibit metals from reaching microorganisms (Oliver and Cosgrove 1974).

The highest KT value for Sb removal was adsorption (338). A variety of adsorbents have been used to remove Sb; it has been reported that adsorption is most effective with Mn, Fe and Al ligands (Belzile et al. 2001) with high removal efficiencies achieved (85–99 %) as Sb has no preference to bind with organic compounds (Guo et al. 2009). Adsorption is therefore most suitable for Sb removal from effluent streams at WWTWs (Ungureanu et al. 2015). More specifically, at the site studied, granulated ferric hydroxide (GEH) is suggested for implementation. This is a relatively new adsorbate, developed at the Department of Water Quality Monitoring (Berlin University), and has been shown to effectively remove Sb to levels below 5 μg/L (Ilavskỳ 2008).

## Conclusions

- Fate and removal of Hg at the works was influenced by the positive correlations observed between this metal and SS as well as COD removal rates. This association results from the high-sorption characteristics of this metal.
- Antimony had a weak interaction with suspended particles in wastewater treatment and was therefore not affected by SS removal at the works.
- Elevated final effluent Sb concentrations compared with crude values was suggested to result from Sb being imported with sludge to the works. Returned sludge liquors are therefore suggested to be responsible for variable and elevated final effluent Sb concentrations at the works. Sludge returns are also suggested to increase Hg concentrations within the works, reducing Hg removal and increasing final effluent Hg concentrations.
- Operational solutions, specifically enhanced HRT and SRT, are an option to increase Hg and Sb removal but are not applicable for implementation at the works studied due to limited capacity for expansion and high costs of infrastructural development. Whilst treatment technologies deployed on sludge liquors require further development before they can be implemented commercially.
- A variety of technologies are available for additional treatment at the works to remove Hg and Sb to levels below UK (Hg 0.05 μg/L, Sb 5 μg/L) and USA (Hg 0.77 μg/L, Sb 5.6 μg/L) standards. It was determined that chemical techniques, more specifically precipitation, were most suitable for Hg removal, whilst adsorption was selected as the most appropriate for Sb removal, in particular, the use of the adsorbate GEH.
